# Development and Testing of a Mobile Phone App for Risk Estimation of Gas Volume Expansion and Intraocular Pressure Elevation in Patients With Intravitreous Gas or Air Tamponade: Interobserver Assessment Study

**DOI:** 10.2196/14592

**Published:** 2019-06-26

**Authors:** Zhaotian Zhang, Fei Li, Haochuan Zhang, Zhipeng Miao, Yantao Wei, Li Wang, Shaochong Zhang

**Affiliations:** 1 State Key Laboratory of Ophthalmology Zhongshan Ophthalmic Center Sun Yat-sen University Guangzhou China; 2 School of Automation, Guangdong University of Technology Guangzhou China

**Keywords:** intraocular pressure, mobile phone, vitrectomy, air

## Abstract

**Background:**

Pars plana vitrectomy (PPV) with intravitreous tamponade of gas or air has been widely used for a series of vitreoretinal diseases. It is estimated that 100,000 patients per year undergo PPV globally, and half of them were subsequently tamponaded with gas or air. According to Boyle’s law (P_1_V_1_=P_2_V_2_), patients with an intravitreous remnant of gas or air will be under high risk of intraocular pressure (IOP) elevation and subsequent vision loss owing to the expanded intravitreous gas or air when traveling post operation to a place with a significantly higher altitude. We always explain to patients why postoperative travel is potentially risky. Emergency cases of elevated IOP caused by postoperative traveling would sometimes come to surgeons. However, there have been few disease education or reference tools for both the surgeons and patients to have better communication.

**Objective:**

The aim of this study was to introduce and evaluate a mobile phone app developed by surgeons (the authors) for preliminary risk estimation of volume expansion and IOP elevation in patients with intravitreous gas or air when traveling to a place of higher altitude.

**Methods:**

The app was developed on the iOS and Android operating systems. Boyle’s law (P_1_V_1_=P_2_V_2_) was the theoretical basis of the app. Intravitreous gas or air volume and altitude values were independent factors to deduce the risk report. Consecutive patients underwent vitrectomy, and those with an intravitreous remnant of gas or air were recruited. The surgeons judged the vertical height of the fluid/gas interface through the dilated pupil; the patients were instructed to judge it according to their visual field when looking straight ahead and line it out on a chart included in the app. Finally, all the patients were required to fill a Likert scale–based questionnaire with 2 main items to evaluate the participants’ user experience and attitudes toward the app.

**Results:**

A total of 50 patients were included (30 males and 20 females). All patients could independently operate the app to complete the test. The median heights of the fluid/gas interface independently judged by the surgeon and patients were 40% (range: 10%-75%) and 41% (range: 9%-78%), respectively (*P*=.63). The median altitude of the participants’ destinations was 150.0 m (range: 0-3490 m). The Bland-Altman analysis revealed a good agreement between the surgeons’ and patients’ judgments (bias of −0.3%), with 95% limits of agreement of −5.8% to 5.3%. Overall, the Likert scale revealed a positive attitude from the patients toward the app.

**Conclusions:**

The app is reliable for patients to have preliminary risk estimation of intravitreous gas or air volume expansion and IOP elevation if travel to a place of higher altitude is planned. The surgeons could also use it as a platform for better disease communication.

## Introduction

Intravitreous gas or air is widely used by vitreoretinal surgeons to achieve anatomic resolution and thus help patients to regain vision [1,2]. Pars plana vitrectomy (PPV) with an intravitreous air/gas tamponade has significantly improved the surgical treatment outcomes for some vitreoretinal diseases, such as rhegmatogenous retinal detachment, macular holes, vitreomacular traction, and foveal retinoschisis [3-6]. Surgeons should always be aware of the effect of intraocular gas or air on the fluctuation of intraocular pressure (IOP). Patients with intravitreous gas or air have generally been advised not to travel in airplanes or travel to locations at significantly higher altitudes as discomfort and even vision loss owing to sharply elevated IOP have been reported [7-11]. The gas or air is tightly sealed within the eyewall, and the eyeball is a semirigid container. Therefore, when the operated eye filled with gas or air is exposed to lower atmospheric pressure, the volume of the intravitreous gas expands. Boyle’s law (P_1_V_1_=P_2_V_2_) provides the theoretical basis for noticing and explaining the potential risks when patients are exposed to different atmospheric pressures [12].

Vitreoretinal surgeons sometimes need to have better communication with their postoperative patients when intravitreous gas or air is present [7-12]. The mathematical models of eyes with intravitreal gas bubbles developed by Amini et al [13-15] and Wong et al [16] were significant and inspired vitreoretinal surgeons to gain an in-depth understanding of the impact of altitude-induced IOP changes. However, there has not been any easily available reference tool for both surgeons and patients to get a preliminary estimation of the risk of volume expansion and IOP elevation when traveling to a place at a higher altitude. In addition, patients with intravitreous gas or air have few reliable tools for self-monitoring the volume after being discharged from the hospital when their surgeons’ professional advice may not be readily available.

The current trend shows that the global ownership rate of mobile phones is quite high [17]. The usefulness of mobile phones has been demonstrated in some medical disciplines because of the rapid advancement of mobile phone technology. Some physicians have tried to use mobile phones and tablets to facilitate their work and have achieved positive outcomes in subspecialties of public health, psychology, oncology, medical education, and so forth [18-30]. Therefore, we hypothesized that mobile phones would be an ideal platform for vitreoretinal surgeons to perform risk estimation and patient education of intravitreous gas or air expansion. To that end, we developed a mobile app based on Boyle’s law and previously reported mathematical models [8,12-16,31]. This study was designed to test the applicability of the app in a real-world setting and evaluate the interobserver agreement on gas volume estimation between surgeons and patients using the app.

## Methods

### Design Principle of the App

The app was developed in the iOS operating system (iOS 12.2; Apple Inc) and Android operating system (Android 8.1; Google LLC) using the programming languages Objective-C (Xcode 10.1; Apple Inc) (for iOS version) and Java (Oracle Java ME SDK 8.2; Oracle Inc) (for Android version), respectively. The app was installed and verified on an iOS mobile phone (iPhone 7+; Apple Inc) and an Android mobile phone (BlackBerry KEY^2^; BlackBerry Limited) as appropriate. The app, named *Intraocular Gas*, has been released by the authors and can be downloaded for free from Apple’s App Store (Apple Inc) and the Google Play app store (Google LLC).

Boyle’s law (P_1_V_1_=P_2_V_2_) was the principle used to measure the prediction accuracy of gas expansion. The calculation depended on gas volume and altitude values. The vitreous cavity was set to be an oblate spheroid (whole volume=4.5 ml). The app calculated the intravitreous gas or air volume based on the vertical height of the fluid/gas interface (in percentages) when the patient was in a sitting position with the head in a level position. Finally, the ratio (expanded volume of intravitreous gas(air)/normal volume of the anterior chamber) was transferred by the app to report a final risk estimation of IOP elevation provided that the patient was transported directly and immediately to a place of higher altitude. The governing equations of the app are illustrated in detail (see Multimedia Appendix 1).

### Interobserver Assessment

**Figure 1:**
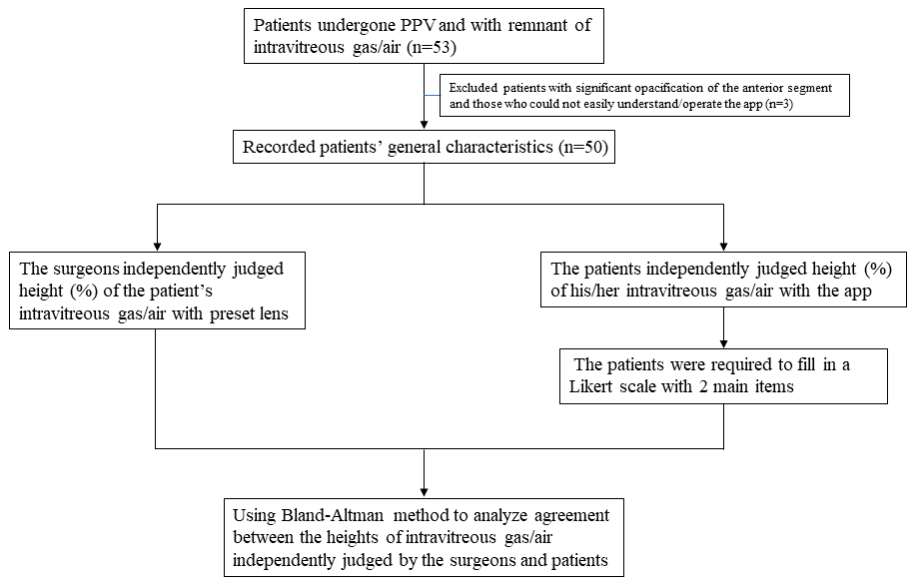
Flowchart for study participants and analysis of the agreement between the heights of the fluid/gas interface independently judged by the surgeons and patients. PPV: pars plana vitrectomy.

The study was approved by the Institutional Review Board of Zhongshan Ophthalmic Center of Sun Yat-sen University (reference number: 2019KYPJ059) and performed in accordance with the World Medical Association’s Declaration of Helsinki. Written informed consent was obtained from each participant. Consecutive patients who had undergone PPV and had an intravitreous remnant of gas or air were recruited. Patients with significant opacification of the anterior segment and those who could not easily understand or operate the app were excluded (Figure 1).

**Figure 2:**
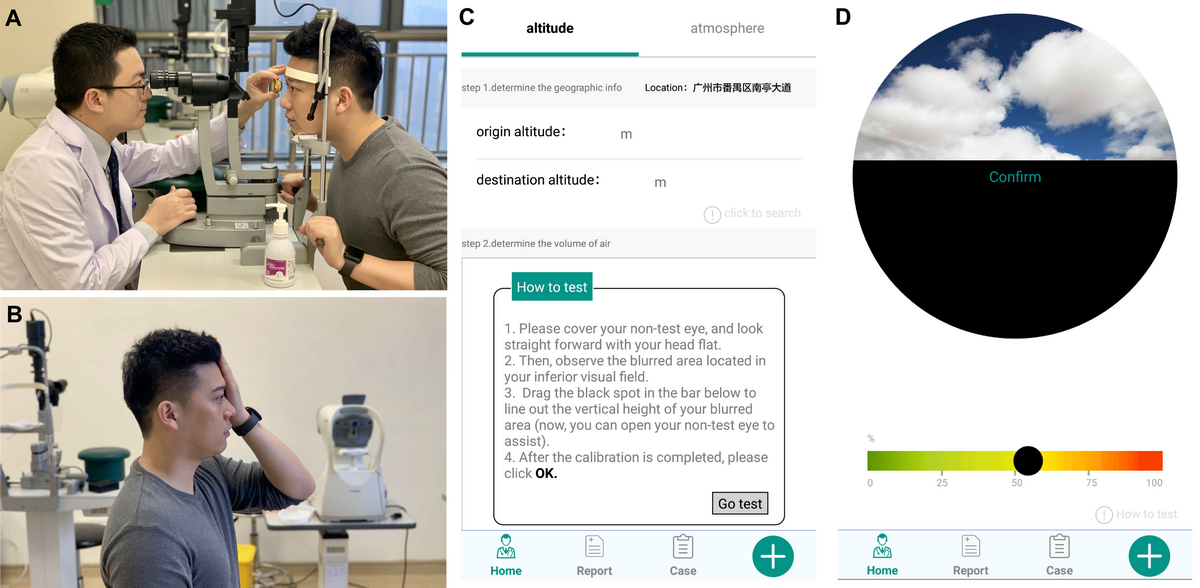
Brief illustration of the interobserver judgement study and usage of the app The surgeon observed the horizontal gas/fluid interface with a preset lens through the dilated pupil and judged the height of it according to the anatomic landmarks (A); With the fellow eye covered, the patient was instructed to judge the height of gas/fluid interface according to his/her visual field when looking straight forward (B); Screenshot of the app showing the brief instruction on how to line out the height of gas/fluid interface in his/her tested eye (C); The chart included in the app for the patient to line out the gas/fluid interface according to his/her judgement (D).

First, the surgeons observed the horizontal fluid/gas interface with a preset lens through the dilated pupil and judged its height according to the anatomic landmarks (fovea, vascular arcades, and optic disc; Figure 2).

Second, with the fellow eye covered, the patient was instructed by the nurses to assess the vertical height of the fluid/gas interface according to his/her visual field when looking straight ahead (Figure 2). The app includes a chart for the patient to line out the interface according to his/her vision (Figure 2). A live demonstration video illustrates the entire procedure on how patients used the app, following the nurse’s brief instruction.(see Multimedia Appendix 2).

**Figure 3:**
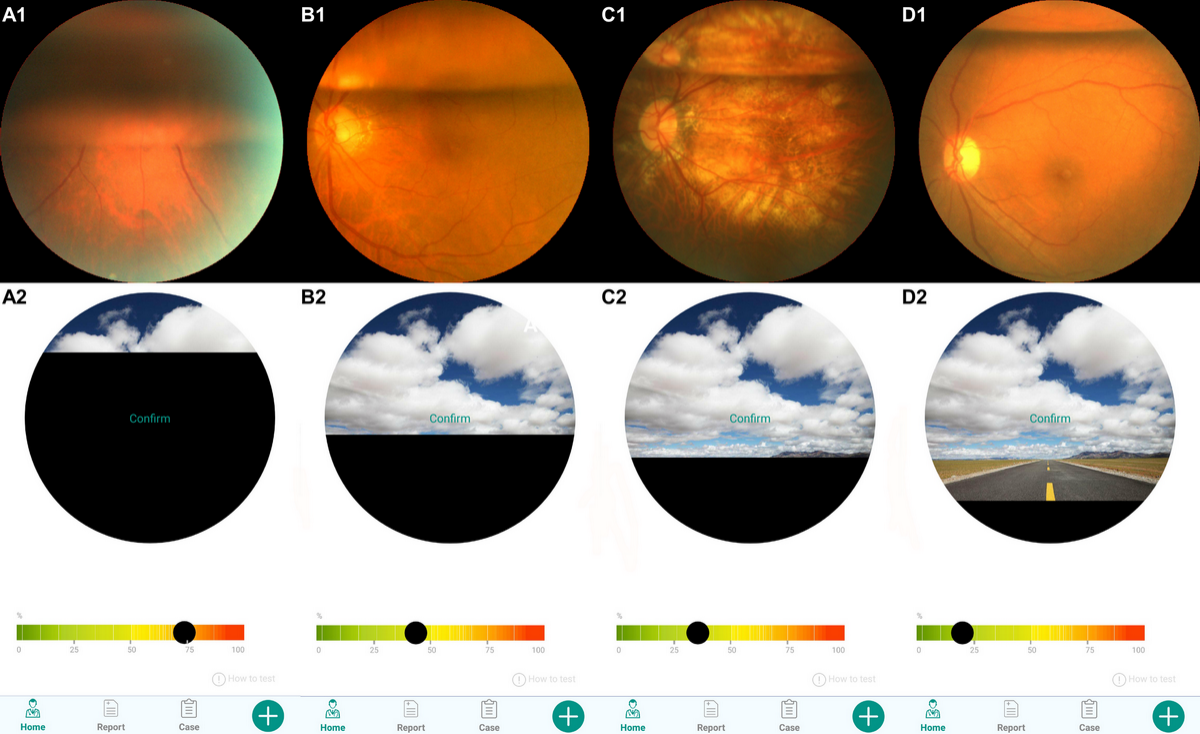
Representative fundoscopy photographs and the corresponding heights of gas/fluid interface independently judged by the surgeons and patients. The top row (A1, B1, C1, and D1) lists fundoscopy photographs of the patients, the height of gas/fluid interface judged by the surgeons were 75%, 45%, 35%, and 20%, respectively (from left to right). The bottom row (A2, B2, C2, and D2) lists corresponding heights of the gas/fluid interface lined out in the app’s chart independently by the patients, the values were 76%, 44%, 35%, and 18%, respectively (from left to right).

Fundus photography was obtained from each patient to verify the height of the fluid/gas interface. Representative fundoscopy photographs and the corresponding heights of the horizontal fluid/gas interface independently judged by the surgeons and patients are illustrated in Figure 3.

Finally, all of the patients were required to fill a Likert scale with 2 items to assess the usability of the app (*Item 1: I think the app is reliable for me to have a postoperative travel plan to another place at a different altitude; Item 2: I will recommend the app to other patients with the same condition as me; 1=strongly disagree, 2=disagree, 3=neutral, 4=agree, 5=strongly agree*).

### Statistical Analysis

Statistical analysis was performed using MedCalc Statistical Software, version 18.9 (MedCalc Software bvba). Continuous variables are expressed as mean (SD) and median (range) according to their distributions (Shapiro-Wilk normality test). Categorical variables are described as proportions (%). Agreement between the heights of the fluid/gas interface independently judged by the surgeons and patients was assessed using the Bland-Altman method with 95% limits of agreement. A *P* value of less than .05 was considered statistically significant.

## Results

### Patient Characteristics

A total of 50 consecutive patients were included (30 males and 20 females; median age: 51.5 years [range: 18-70 years]; visual acuity ranged from counting fingers [CF] to 20/25). All the patients underwent surgery in the Zhongshan Ophthalmic Center of Sun Yat-sen University (Guangzhou, China; altitude=10 m). All the patients could easily and independently operate the app to complete the test following the nurse’s brief instructions.

### Outcomes of Interobserver Assessment

The median heights of the fluid/gas interface independently judged by the surgeon and patients were 40% (range: 10%-75%) and 41% (range: 9%-78%), respectively (with no significant statistical difference when analyzed with the Wilcoxon matched-pairs signed rank test; *P*=.63). The median altitude of the participants’ places of planned destination was 150.0 m (range: 0-3490 m). The median expanded volumes of intravitreous gas or air independently judged by the surgeons and patients and calculated by the app was 0.02 ml (range: 0-2.4 ml) and 0.02 ml (range: 0-2.3 ml), respectively (with no significant statistical difference when analyzed with the Wilcoxon matched-pairs signed rank test; *P*=.55). In total, 38% (19/50) of the patients planned to travel back by airplane, 30% (15/50) by train (high-speed/ordinary=11/4), and the remaining 32% (16/50) by car/bus. The main characteristics of all the patients are summarized into a table in Multimedia Appendix 3).

**Figure 4:**
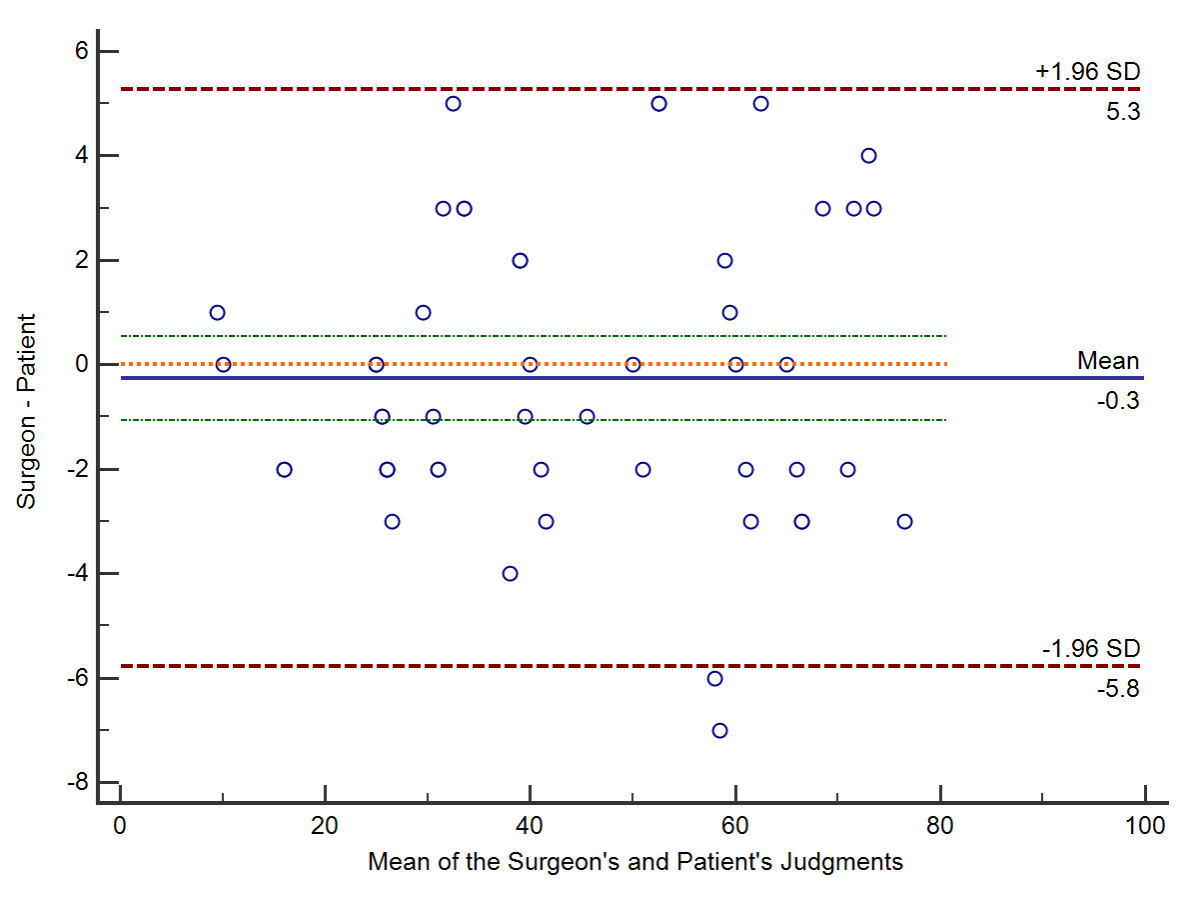
The Bland-Altman analysis revealed a bias of −0.3% (solid line) between the surgeons’ and patients’ judgements, with 95% limits of agreement, of −5.8% to 5.3% (dash lines).

**Figure 5:**
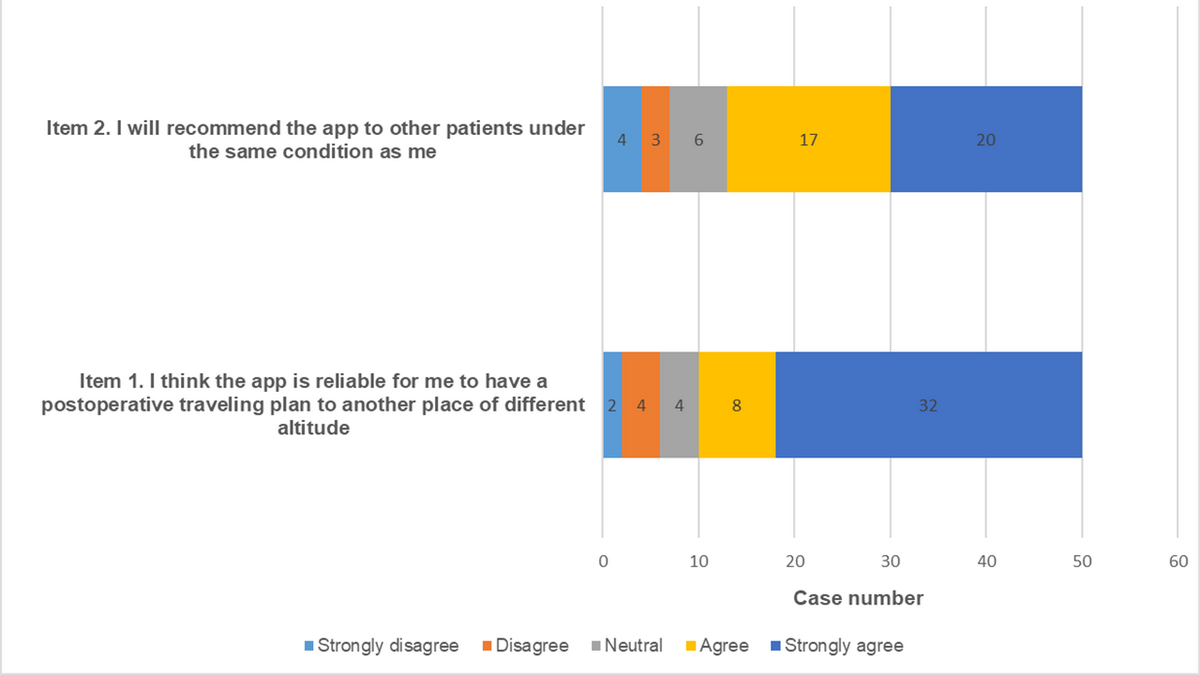
The Likert scale revealed a generally positive attitude from the patients toward the app.

The Bland-Altman analysis revealed a bias of −0.3% between the surgeons’ and patients’ judgments, with 95% limits of agreement, of −5.8% to 5.3% (Figure 4). The Likert scale revealed a generally positive attitude from the patients toward the app (Figure 5).

## Discussion

In this study, we demonstrated the design principle and clinical applications of a mobile app developed by the authors, and we showed the app’s potential in preliminary risk estimation of volume expansion and IOP elevation in patients with an intravitreous gas or air tamponade. To the best of our knowledge, the app is the first one developed by vitreoretinal surgeons to solve the actual conundrum of intravitreous gas or air in our daily clinical work.

### Theoretical Basis and Design Principle of the App

The essential factors of the app include the following: the mechanics and kinetics of intravitreous gas or air tamponading after PPV; the semirigid property of the eyeball; Boyle’s law (pressure × volume=constant); the relationship between atmospheric pressure and altitude; and the ratio of the expanded volume of intravitreous gas or air to that of the anterior chamber. As proposed by Lincoff and Aronowitz, the eyeball has 3 compensation mechanisms to accommodate the expansion of intravitreous gas or air: choroidal compression, scleral expansion, and accelerated aqueous outflow [11,32]. These mechanisms are significant for us to have an in-depth understanding of elevated IOP induced by decreases in atmospheric pressure.

Unlike previously reported mathematical models [13-15,33], we developed our app based on the assumption that the patient with intravitreous gas or air would be traveling immediately from one place to another. Therefore, the abovementioned compensation mechanisms were not incorporated into the app design. Another reason for developing this app was to provide a readily available electronic tool for both surgeons and patients to calculate a preliminary estimation of the risk. Therefore, it was decided to develop the app based on a simplified and feasible principle. We contributed our best efforts to making the app suitable for both surgeons and patients to achieve more friendly interactions and higher interobserver agreement.

### The App’s Potential to Help Disease Communication and Preliminary Risk Estimation

There have been continuous reports on the risks of postoperative travel for vitrectomied eyes before the intravitreous gas or air is completely absorbed [7,8,34,35]. We referred to Boyle’s law to explain the phenomenon, which is also the governing equation incorporated into this app. On the basis of previous studies of animal models, simulated flight experiments, mathematical models, and case reports, patients are generally advised against traveling through lower atmospheric pressure environments [7,9,10,14,33-37]. Patients are exposed to lower atmospheric pressure when they embark on postoperative travel by airplane or by car/train to areas with higher altitudes. A previous animal study by Dieckert et al [37] indicated that it is unsafe to fly with intraocular gas of any volume, unless cabin pressurization can be maintained at 2000 feet at 706 mmHg or higher. However, there is still some controversy on the seriousness of this risk because the cost of vision loss is huge and irreversible, even though the risk is theoretically low.

In our clinical work, we always explain to patients why postoperative travel is potentially risky. Emergency cases involving elevated IOP caused by postoperative travel sometimes came to our clinic, and there are anecdotal reports and authors’ experience to support the existence of this risk. There are 4 possible underlying causes of the vision-threatening tragedy: absence or inadequate disease education regarding the risk of postoperative travel with an intravitreous gas or air tamponade; lack of an easily understandable education tool for surgeons and patients to engage in more effective interaction about the risk of gas or air expansion as the risk is informed according to the physical law, mathematical models, and case reports; lack of postoperative monitoring by surgeons of the volume of intravitreous gas after the patient is discharged from the hospital; and the fluke mind of some patients who lack sufficient awareness of the risk of expanded intravitreous gas or air [8]. This app can enable both surgeons and patients to better keep track of the risk of expanded intravitreous gas or air.

### Where and When the App Will Serve Surgeons and Patients

We speculate that the app will be practical in many areas of the world. Indeed, previous reports indicate that significant elevation differences between 2 places should be considered a risk factor for elevated IOP in such patients, regardless of the mode of transportation they choose [7,8,10,34,35]. The app directly offers a preliminary evaluation of the risk of IOP elevation based on 2 independent factors, namely, the height of the intravitreous gas or air and the altitude. According to the data accessed from Google Earth, many large cities (especially urban areas) have significantly lower altitudes than smaller towns (especially rural and mountain areas). Medical institutions are usually located in large cities, so some patients may be discharged from the hospital to a higher altitude or to even travel by an airplane. Thus, it is likely that such problems with intravitreous gas or air expansion exist in many ophthalmic institutes around the world.

In China, as the land area is large, different cities are located at different altitudes and have different atmospheric pressure. When patients travel from one place to another at a higher altitude, they are exposed to a higher risk of gas expansion and IOP elevation. In our institute (one of the national tertiary referral centers; Zhongshan Ophthalmic Center of Sun Yat-sen University, Guangzhou, China; altitude=10 m), approximately 30% of our patients who receive vitreoretinal surgeries come from other provinces that generally are at higher altitudes. For instance, in this study, one patient was from the city of Lhasa (altitude=3490 m), 3 were from the city of Guiyang (altitude=1277 m), and 5 were from the city of Kunming (altitude=1842 m). We can imagine that there is a risk of elevated IOP if patients with intravitreous gas or air have postoperative travel back home.

### High Interobserver Agreement Enhances the App’s Potential for Patients to Self-Monitor

The app can provide a reliable reference tool for patients to do self-monitoring and generate a preliminary risk report. The significant advantages of the app can be summarized into the following aspects: acceptable accuracy of the estimation as this study revealed high interobserver agreement between the surgeons and patients; the app could serve as a physician-patient communication platform as they share the same logistics for calculating the risk of expanded intravitreous gas or air; and high acceptability for the patients as the Likert scales revealed a significantly positive attitude from the patients toward the app because it is user-friendly, and it is not difficult for patients to understand the underlying principles.

To the best of our knowledge, this study is the first one to have interobserver analyses of the height of the intravitreous fluid/gas interface independently judged by surgeons and patients. Inspired by previous studies [16,31], we designed the app from 2 perspectives: the surgeons’ professional judgment with dilated fundoscopy and patients’ true vision perception. The surgeon directly observed the horizontal fluid/gas interface with a preset lens through the dilated pupil and judged the vertical height of the fluid/gas interface according to anatomic landmarks. It is not difficult for surgeons to make a reliable judgment of the height; however, there are few guidelines for patients to express their personal judgments, which decreases patients’ awareness and limits the efficacy of disease education. The app eliminated this barrier and imbalance with its modified design.

The app is the first one designed based on the patients’ true vision perception with intravitreous gas or air. Owing to the great differences in the refraction index, the intravitreous fluid and gas or air produces vastly different vision results for the patients. Intravitreous gas located in the upper part of the vitreous cavity makes vision darker and more blurred, whereas intravitreous fluid located in the lower part of the vitreous cavity makes vision almost normal. According to the law of photorefraction through the visual axis, clear vision (through the intravitreous fluid) is in the patient’s upper visual field and blurred vision (through the intravitreous gas) is located in the patient’s lower visual field. Therefore, a patient can easily line out the fluid/gas interface according to the 2 different visions in his/her operated eye. We are encouraged by the high interobserver agreement and believe that the app will be a reliable reference tool for patients in case professional advice from their surgeons is unavailable. In addition, the app is worth recommending to our vitreoretinal colleagues as a supplementary tool. It is important to teach patients how to evaluate their postsurgical conditions. In the app, gas volume estimation can be done easily either by retinal surgeons or by the patients themselves. As altitude information can be objectively obtained from the internet, the accuracy of gas expansion estimation will mainly be affected by gas volume estimation. According to the results, gas volume estimation by surgeons and patients themselves achieved good agreement, which means the patients are more likely to make correct estimations of gas volume through self-evaluation with the app. It is well known that patients usually have lower visual acuity after PPV with a gas or air tamponade, when the visual axis is blurred by the gas or air. Therefore, we also evaluated whether patients with lower visual acuity (finger counting or hand movement) could accurately estimate their intravitreous gas volume. The results showed that even in these patients, their self-estimations were quite close to those of the surgeons, which means the app will be applicable for different types of patients.

### Limitations and Recommendation for Future Refinement of the App/Research

There are several limitations that should be acknowledged. The sample size was relatively small, so the efficacy of the app needs to be tested in a larger population. The compensation mechanisms of the eyeball to accommodate for the expansion of intravitreous gas or air under decreased atmospheric pressure were not included in the app design, which could decrease the validity of risk estimation. The volume of the vitreous cavity is set to 4.5 ml, which may not be completely consistent in some patients, such as those with hyper-myopia. In vivo experiments on humans or animals were not performed to verify the true accuracy of the risk estimation.

Although certain limitations exist in this study, the app is a reliable reference tool for both surgeons and patients to make a preliminary estimation of intravitreous gas or air expansion and IOP elevation. Furthermore, the good interobserver agreement and user-friendly interface provide a readily available platform to create better physician-patient interaction with regard to the risk of expanded intravitreous gas or air after surgery. Further refinement of the app and in vivo experiments are warranted to provide more accurate risk estimation for surgeons and patients.

## Appendix

Multimedia Appendix 1 The governing equations of the app.

Multimedia Appendix 2 Video demonstration of the app’s usage.

Multimedia Appendix 3 The main characteristics of all the patients.

## Abbreviations

IOP: intraocular pressure
PPV: pars plana vitrectomy
