# Supplementary material for: Development and Testing of a Mobile Phone App for Risk Estimation of Gas Volume Expansion and Intraocular Pressure Elevation in Patients With Intravitreous Gas or Air Tamponade: Interobserver Assessment Study
Source: JMIR Mhealth Uhealth. 2019 Jun 26;7(6):e14592. doi: 10.2196/14592 (PMC6617918; doi:10.2196/14592)
Supplement: Multimedia Appendix 1 [file mhealth_v7i6e14592_app1.pdf]

Multimedia Appendix 1

The governing equations of the APP were developed in steps, as described below:

How to determine the volume of intravitreous gas/air[16]

1. The vitreous cavity is set to be an oblate spheroid, the entire volume of which is 4.5 ml. The brief calculation is as follows:

$$V_1 = (4/3)\pi a^2 b \tag{1}$$

Where V_1=volume (mm^3) of the vitreous cavity = 4,500 mm^3 = 4.5 ml, a = length of major semiaxis (mm) = horizontal eye axis/2 ≈ 22 /2 = 11 mm, and b=length of minor semiaxis (mm)=vertical eye axis/2 ≈ 18/2 = 9 mm.

2. The vertical height of the intravitreous gas is estimated by the physician (when at the in-patient department or clinic) or the patient by him/herself (for self-assessment or when the physician's instructions are unavailable) when the patient is in a sitting position with the head held level.

   Surgeon's estimation method: The surgeon directly observes the horizontal fluid/gas interface with a preset lens through the dilated pupil and judges the vertical height of the fluid/gas interface according to anatomic landmarks (fovea, vascular arcades, optic disc, etc.). The height of the fluid/gas interface is expressed as a percentage of the vertical diameter of the vitreous cavity (e.g., when the interface is at the level of the fovea, the percentage of the interface is 50%; when at the middle of the superior vascular arcade, the percentage is about

40%. The surgeon estimates the volume according to his or her expertise and experience.

Patient's estimation method: The patient judges the horizontal fluid/gas interface when he/she is looking straight forward. Because of the great differences in the refraction index, the intravitreous fluid and gas/air produce greatly different vision results for the patient (as shown in Figure 2). Intravitreous gas located in the upper part of the vitreous cavity makes the vision darker and more blurred while intravitreous fluid located in the lower part of the vitreous cavity makes vision nearly the same as normal. According to the law of photorefraction through the visual axis, clear vision (through the intravitreous fluid) is located in the patient's upper visual field, and blurred vision (through the intravitreous gas) is located in the patient's lower visual field. Therefore, the patient can easily line out the fluid/gas interface according to the two different vision types in his/her operated eye. With the fellow eye covered, the patient is instructed to assess the height (in percentages) of the fluid/gas interface according to his/her visual field when looking straight ahead. The APP includes a chart for the patient to easily line out the interface according to his/her vision (Figure 1).

3. Estimation of the volume of intravitreous gas/air according to the height (in percentages) of the fluid/air interface

As the vitreous cavity is set to be oblate spheroid, the equation below can be used to deduce the volume of intravitreous gas, using the height of the fluid/gas interface as the only independent factor.

$$V_2 = V_1 (3P^2 - 2P^3) \quad (2)$$

Where V_1 = volume (mm^3) of the vitreous cavity = 4,500 mm^3 = 4.5 ml, V_2 = volume of intravitreous gas (mL), and P = height (percentage) of the fluid/gas interface in the vitreous cavity ($0 \leq P \leq 1$).

How to determine atmospheric pressure[12]

We decided to estimate atmospheric pressure according to a simplified equation, using different cities' altitudes as the only independent factor (with every 12 m decrease in altitude, the corresponding atmospheric pressure decreases 1 mmHg). We set the atmosphere pressure at sea level to 760 mmHg. Thus, the atmospheric pressure in other places can be calculated according to the following equation:

$$\text{atmospheric pressure} = 760 - (\text{the corresponding altitude (m)} / 12)\ \text{mmHg} \quad (3)$$

The geographic elevations for different cities are located from Internet data (Wikipedia and Google Earth). The APP also permits patients to enter an accurate geographic elevation of his/her interested place (such as a high mountain or building) in order to make it more adaptable to different circumstances.

How to determine the expansion volume of the intravitreous gas[12,16]

The expansion volume of intravitreous gas can be determined by Boyle's Law ($P_1V_1=P_2V_2$). Following this physical law, it is possible to deduce how much the intravitreous gas bubble will expand to make the IOP normal again.

If we set the patient's normal IOP at the two places to be the same as 16 mmHg, the extreme pressure of the intravitreous gas=atmospheric pressure at the corresponding position+16 mmHg.

According to Equation 3 and Boyle's Law, we can calculate the ratio of expansion from the following:

V_4 / V_3 = extreme pressure of the intravitreous gas at Place 1 / extreme pressure of the intravitreous gas at Place 2

= [760 – (corresponding altitude at Place 1 (m)/12) + 16] / [760– (corresponding altitude at Place 2 (m)/12) + 16]

= [9312−altitude at Place 1 (m)] / [9312−altitude at Place 2 (m)]

Where V_3=volume (ml) of intravitreous gas at Place 1 and V_4=volume (ml) of intravitreous gas at Place 2

Thus, the theoretical expansion volume of intravitreous gas when the patient moves from Place 1 to Place 2 is as follows:

$$V_{expansion} = V_4 - V_3 = 4.5\ (3P^2 - 2P^3)\ (A_2 - A_1) / (9312 - A_2)$$

Where $V_{expansion}$ = the theoretical expanding volume (ml) of intravitreous gas when the patient moves from Place 1 to Place 2, P = height (percentage) of fluid/gas interface in the vitreous cavity when the patient is at Place 1 ($0 \leq P \leq 1$), A_1 = corresponding altitude (m) at Place 1, and A_2 = corresponding altitude (m) at Place 2.

How to estimate the risk of gas expansion

First, we set the volume of the anterior chamber to 0.25 ml. Considering the compensation mechanism of the eye when the intravitreous gas expands, we define

that risk into 5 grades (1 = very low risk; 5 = extremely high risk). We define the risk according to the ratio of the expansion volume of the intravitreous gas to the volume of the anterior chamber, that is:

risk ratio = $V_{expansion}$ / 0.25

= 18 $(3P^2 – 2P^3) (A_2 – A_1) / (9312 – A_2)$

Where, as in Equation 3, P=height (percentage) of fluid/gas interface in the vitreous cavity when the patient is at Place 1 ($0 \leq P \leq 1$), A_1 = corresponding altitude (m) at Place 1, and A_2=corresponding altitude (m) at Place 2.

We define the risk according to the risk ratio as follows:

low risk: risk ratio < 1/5

medium risk: 1/5 ≤ risk ratio < 2/5

high risk: 2/5 ≤ risk ratio < 4/5

very high risk: 4/5 ≤ risk ratio < 1

extremely high risk: risk ratio ≥ 1

For example, we can explain why patients may suffer from acute ocular eye pain and vision loss when they leave Guangzhou (Guangdong Province, China) and go to Kunming (Yunnan Province, China). The altitudes at Guangzhou and Kunming are 10 m and 1894 m, respectively. If we assume the height of a patient's fluid/gas interface in the vitreous cavity when he or she left Guangzhou was 20%, we can calculate the risk ratio as follows:

risk ratio = 18 $(3P^2 – 2P^3) (A_2 – A_1) / (9312 – A_2)$

= 18 $(3\times0.2^2 – 2\times0.2^3) (1894-10)/(9312-1894)$

= 0.48 (high risk)

It is obvious that this patient is at high risk of gas expansion.

The APP can be downloaded and installed via the link:

https://play.google.com/store/apps/details?id=com.intraocular&rdid=com.intraocular

The live demonstration of the APP's usage can be referred to the link:

https://youtu.be/U3dd7yUn6Bk
