# Supplementary material for: Development and Testing of a Mobile Phone App for Risk Estimation of Gas Volume Expansion and Intraocular Pressure Elevation in Patients With Intravitreous Gas or Air Tamponade: Interobserver Assessment Study
Source: JMIR Mhealth Uhealth. 2019 Jun 26;7(6):e14592. doi: 10.2196/14592 (PMC6617918; doi:10.2196/14592)
Supplement: Multimedia Appendix 3 [file mhealth_v7i6e14592_app3.pdf]

Table 1. General Characteristics of the Patients in the Study.

Total Patients	n = 50
Male	n = 30 (60%)
Female	n = 20 (40%)
Age (years)	Median = 51.5, IQR[a] = 12
	Min = 18, Max = 70
≤ 30	n = 2 (4%)
31-50	n = 18 (36%)
≥ 51	n = 30 (60%)
Visual Acuity (Snellen)	Median = 0.02, IQR = 1.601 (logMAR[b] unit)
	Min = HM[c], Max = 20/25
≥ 20/40	n = 4 (8%)
< 20/40 and ≥ 20/200	n = 14 (28%)
< 20/200 and ≥ 20/2000	n = 8 (16%)
FC[d]	n = 15 (30%)
HM	n = 9 (18%)
Main Diagnosis	
Retinal detachment	n = 21 (42%)
Macular hole	n = 16 (32%)
Epimacular membrane	n = 6 (12%)
PDR[e]	n = 7 (14%)
Altitude of Destination (m)	Median = 150, IQR = 1238
	Min = 0, Max = 3650
≤ 500	32 (64%)
> 500 and ≤ 1000	4 (8%)
> 1000 and ≤ 2000	11 (22%)
> 2000	3 (6%)
Travel Vehicle after Discharge	
Airplane	n = 19 (38%)
Train (high speed/ordinary)	n = 15 (30%; 11/4)
Car/bus	n =16 (32%)

[a]IOR: interquartile range.

[b]logMAR: logarithm of the minimum angle of resolution.

[c]HM: hand motion.

[d]FC: finger counting.

[e]PDR: proliferative diabetic retinopathy.
